# Effects of radiation therapy on tissue and serum concentrations of tumour associated trypsin inhibitor and their prognostic significance in rectal cancer patients

**DOI:** 10.1186/1748-717X-6-100

**Published:** 2011-08-24

**Authors:** Alexander Gaber, Christina Stene, Kristina Hotakainen, Björn Nodin, Ingrid Palmquist, Anders Bjartell, Ulf-Håkan Stenman, Bengt Jeppsson, Louis B Johnson, Karin Jirström

**Affiliations:** 1 Department of Clinical Sciences, Division of Pathology, Lund University, Skåne University Hospital, Lund, Sweden; 2 Department of Clinical Sciences, Division of Colorectal Surgery, Lund University, Skåne University Hospital, Malmö, Sweden; 3 Department of Clinical Chemistry, University of Helsinki and Helsinki University Central Hospital, Helsinki, Finland; 4 Center for Molecular Pathology, Department of Laboratory Medicine, Lund University, Skåne University Hospital, Malmö, Sweden; 5 Department of Clinical Sciences, Division of Urological Cancers, Lund University, Skåne University Hospital, Malmö, Sweden

**Keywords:** Rectal cancer, tissue micro array, TATI, radio therapy, prognosis, biomarker

## Abstract

**Background:**

We have previously demonstrated that elevated concentrations of tumour-associated trypsin inhibitor (TATI) in both tumour tissue (t-TATI) and in serum (s-TATI) are associated with a poor prognosis in colorectal cancer patients. It was also found that s-TATI concentrations were lower in patients with rectal cancer compared to patients with colon cancer. In this study, we investigated the effects of neoadjuvant radiotherapy (RT) on concentrations of t-TATI and s-TATI in patients with rectal cancer.

**Methods:**

TATI was analysed in serum, normal mucosa and tumour tissue collected at various time points in 53 rectal cancer patients enrolled in a case-control study where 12 patients received surgery alone, 20 patients 5 × 5 Gy (short-term) preoperative RT and 21 patients 25 × 2 Gy (long-term) preoperative RT. T-TATI was analysed by immunohistochemistry and s-TATI was determined by an immunofluorometric assay. Mann-Whitney U test and Wilcoxon Z (Z) test were used to assess t-TATI and s-TATI concentrations in relation to RT. Spearman's correlation (R) test was used to explore the associations between t-TATI, s-TATI and clinicopathological parameters. Overall survival (OS) according to high and low t-TATI and s-TATI concentrations was estimated by classification and regression tree analysis, Kaplan-Meier analysis and the log rank test.

**Results:**

RT did not affect concentrations of t-TATI or s-TATI. In patients receiving short-term but not long-term RT, s-TATI concentrations were significantly higher 4 weeks post surgery than in serum drawn prior to surgery (Z = -3.366, P < 0.001). T-TATI expression correlated with male gender (R = 0.406, P = 0.008). High t-TATI expression in surgical specimens was associated with a significantly shorter OS (P = 0.045). S-TATI concentrations in serum drawn at all time points were associated with an impaired OS (P = 0.035 before RT, P = 0.001 prior to surgery, P = 0.043 post surgery). At all time points, s-TATI correlated with higher age (P < 0.001-0.021) and with increased s-creatinine concentrations assessed prior to surgery (P = 0.041).

**Conclusions:**

The results presented here further validate the utility of t-TATI and s-TATI as prognostic biomarkers in patients with rectal cancer, independent of neoadjuvant RT.

## Background

We have previously demonstrated that tumour-associated trypsin inhibitor (TATI), also called pancreatic secretory trypsin inhibitor (PSTI) and serine protease inhibitor Kazal type 1 (SPINK1), is a biomarker of poor prognosis in colorectal cancer patients, both as assessed in tumour tissue (t-TATI) [1] and in serum (s-TATI) [2], whereby the strongest independent prognostic value was seen for s-TATI [2]. While there was no association between t-TATI and tumour location, s-TATI concentrations were significantly lower in patients with rectal cancer compared to those with colon cancer [2]. This could be due to biological differences between colonic and rectal tumours, but as the majority of the rectal cancer patients in the study (85/107) had received neoadjuvant radiotherapy (RT), we could not exclude the possibility that RT affects s-TATI concentrations in rectal cancer patients.

For patients with rectal cancer, preoperative RT has been found to significantly reduce the risk for local recurrence and death [3,4]. Studies on the effects of RT on rectal tumour tissue have shown that tumour cells become swollen and that the stromal compartment acquires an abundance of fibroblasts, granulocytes and lymphocytes [5]. Ionising radiation induces a widespread oxidative damage at the cellular level [6] and has been found to remodel the extracellular matrix (ECM) and affect various enzymes such as transforming growth factor receptor beta 1 (TGF-ß1), matrix metalloproteinase 2 (MMP-2) and MMP-9 [7]. Urokinase-type plasminogen activator (uPA), and other members of the serpin family like plasminogen activator inhibitor 1 (PAI-1), have also been found to play important roles in the remodelling of ECM [5].

Trypsin is a potent matrix serine protease (MSP) that hydrolyses a variety of proteins and activates other MSPs and MMPs [8,9]. TATI is a trypsin inhibitor that balances concentrations of trypsin and also functions as a weak inhibitor of other serine proteinases [10,11]. In addition, TATI has been found to be involved in tissue repair *in vitro *[12] and to play an important role in the tumour microenvironment and tumour cell invasion [13]. In the light of these findings, it could be hypothesized that RT in rectal cancer affects TATI concentrations in tissue and/or serum and, hence, survival. As we are not aware of any previous studies describing the effects of RT on the tumour-specific expression of TATI or its serum concentrations in any cancer form, the aim of the present study was to investigate whether neoadjuvant RT affects t-TATI and s-TATI concentrations in rectal cancer patients, and to assess their prognostic values. For this purpose, TATI was analysed in serum, non-malignant rectal mucosa and tumour tissue samples taken at different time points; before, during, and after RT, and for serum also 4 weeks after surgery, in a prospective cohort of 53 patients with rectal cancer. Given the previously observed association between higher age and increased s-TATI concentrations [2], we also examined the relationship between t-TATI, s-TATI, age, and the concentrations of s-creatinine and carcinoembryonic antigen (s-CEA) in preoperatively drawn serum samples.

## Methods

### Patients

The study was designed as a case-control study and consisted of 77 patients diagnosed and treated for rectal cancer at Skåne University Hospital, Malmö, between 2003 and 2007. A total number of 24 patients were excluded, 8 for whom the diagnosis was revised to high grade dysplasia, 8 due to an impaired general condition, 3 patients with synchronous tumours in the colon, 4 patients who declined to participate, and 1 patient was excluded due to logistic reasons. Thus, the study cohort comprised 53 patients, 36 (67.9%) males and 17 (32.1%) females. Patients were staged according to the TNM system (American Joint Committee on Cancer, AJCC, 6^th ^edition) [14]. One group received short-term regimen of preoperative RT (25 Gy; n = 20, 37.7%), another group received long-term regimen of preoperative RT (50 Gy, n = 21, 39.6%), and a control group underwent surgery alone (n = 12, 22.6%). Patient and tumour characteristics according to treatment groups are shown in Table 1. Serum and tissue samples were collected from the three different groups at different time points. From non-irradiated patients and the short-term RT group, serum was drawn on three occasions; before RT, after RT (prior to surgery), and at routine follow-up 4 weeks post surgery. In the long-term RT group, serum was collected at two additional occasions; 12 days into RT (at half-time) and after completion of treatment. No patients received neoadjuvant chemotherapy. Thirteen patients (24.5%) received adjuvant chemotherapy after surgery. Serum samples were stored at -20°C until analysis. Tissue biopsies, both from tumour and normal mucosa, were sampled at the same time points as serum, except at follow-up 4 weeks post-surgery (Additional file 1). Tissue samples were formalin fixated and paraffin embedded. The study has been approved by the Ethics committee at Lund University (ref 144/2004 with amendment 597/2006) and written consent was obtained from the patients.

**Table 1:** Patient characteristics

	No RT	Short-term RT	Long-term RT	*p-value*
***n*(%)**	**12**	**20**	**21**	

**Age**				
< 75	8(66.7)	15(75.0)	16(76.2)	
≥ 75	4(33.3)	5(25.0)	5(23.8)	
Missing	0(0)	0(0)	0(0)	*0.823*
				
**Gender**				
male	9(75.0)	14(70.0)	13(61.9)	
female	3(25.0)	6(30.0)	8(38.1)	
Missing	0(0)	0(0)	0(0)	*0.717*
				
**Differentiation grade**				
Well-Moderate	12(100.0)	13(65.0)	17(81.0)	
Low	0(0)	6(30.0)	2(9.5)	
Missing	0(0)	1(5.0)	2(9.5)	*0.107*
				
**T-stage**				
I	0(0)	1(5.0)	1(4.8)	
II	5(41.7)	7(35.0)	4(19.0)	
III	6(50.0)	12(60.0)	11(52.4)	
IV	1(8.3)	0(0)	3(14.3)	
Missing	0(0)	0(0)	2(9.5)	*0.421*
				
**N-stage**				
0	7(58.3)	10(50.0)	11(52.4)	
I	2(16.7)	3(15.0)	7(33.3)	
II	3(25.0)	7(35.0)	1(4.8)	
Missing	0(0)	0(0)	2(9.5)	*0.144*
				
**M-stage**				
0	11(97.7)	18(90.0)	19(90.5)	
I	1(8.3)	2(10.0)	1(4.8)	
Missing	0(0)	0(0)	1(4.8)	*0.752*
				
**Disease stage**				
Stage I	3(25.0)	7(35.0)	4(19.0)	
Stage II	4(33.3)	3(15.0)	7(33.3)	
Stage III	4(33.3)	8(40.0)	7(33.3)	
Stage IV	1(8,3)	2(10.0)	1(4.8)	
Missing	0(0)	0(0)	2(9.5)	*0.628*
				
**Operative procedure**				
Rectum resection	9(75.0)	11(55.0)	9(42.9)	
Rectum amputation	2(16.7)	8(40.0)	8(38.1)	
Hartmann's procedure	1(8.3)	1(5.0)	3(14.3)	
Missing	0(0)	0(0)	1(4.8)	*0.506*
				
**Vascular invasion**				
No	9(75.0)	14(70.0)	14(66.7)	
Yes	3(25.0)	6(30.0)	5(23.8)	
Missing	0(0)	0(0)	2(9.5)	*0.125*

### Tissue microarrays

Two tissue microarray (TMA) series were constructed; one biopsy TMA with 1 × 1 mm cores from biopsies with normal tissue (sampled 2 cm from the tumour) and cancer, respectively, and one TMA with normal and cancerous tissue from the surgical specimens, whereby 2 × 1 mm cores were extracted from areas representing viable, non-necrotic tumour, and adjacent, microscopically benign, rectal mucosa, respectively.

### Immunohistochemistry and staining evaluation

Four-micrometer sections from the TMAs were pre-treated in the DAKO PT-link module using a standard protocol and buffer supplied by the manufacturer. Slides were then stained in a DAKO Autostainer-plus using the EnVision™ FLEX including Peroxidise-Blocking Reagent (DAKO, Glostrup, Denmark) with a TATI monoclonal antibody (6E8) diluted 1:150 as described earlier [15]. In line with previous findings [1], TATI was expressed in the cytoplasm, and the percentage of positive tumour cells was estimated separately in each core. Intensity was annotated using a scale from 0-3. The immunohistochemical staining was evaluated independently twice by one observer (AG). For each case, a mean score from both cores was calculated, as well as, wherever possible, the best score [1].

### Immunofluorometric assay of s-TATI

Samples were analysed using a time-resolved immunofluorometric assay, with the MAb 6E8 as a capture antibody for TATI and a europium (Eu) labelled antibody 11B3 as a tracer [2,15]. Fluorescence was measured with a 1420 VIKTOR2 time-resolved fluorometer (Wallac, Turku, Finland), where the lower limit of detection for TATI was 0.1 μg/L and the measuring range 0.5-90 μg/L.

### Statistical analysis

Comparison of TATI concentrations in tissue and serum at different time points in patient subgroups receiving no preoperative RT, short-term RT and long-term RT was performed using the Mann-Whitney U-test and Wilcoxon Z test (Z). Spearman's correlation (R) test was used to explore the associations between t-TATI and s-TATI concentrations, before and after RT, and clinicopathological parameters, including s-CEA and s-creatinine. Classification and regression tree (CRT) analysis was used to assess optimal cut-offs for t-TATI and s-TATI in relation to OS. Kaplan-Meier analysis and log-rank tests were applied to compare survival in strata according to low and high concentrations of t-TATI and s-TATI derived from the CRT analysis. P-values of < 0.05 were considered significant. Only two-sided results were used. Statistical analyses were carried out using the Statistical Package for Social Sciences, SPSS 16.0 package (SPSS Inc, Chicago, Ill).

## Results

### TATI concentrations in tumour tissue, normal mucosa, and serum in relation to radiotherapy

**Figure 1:**
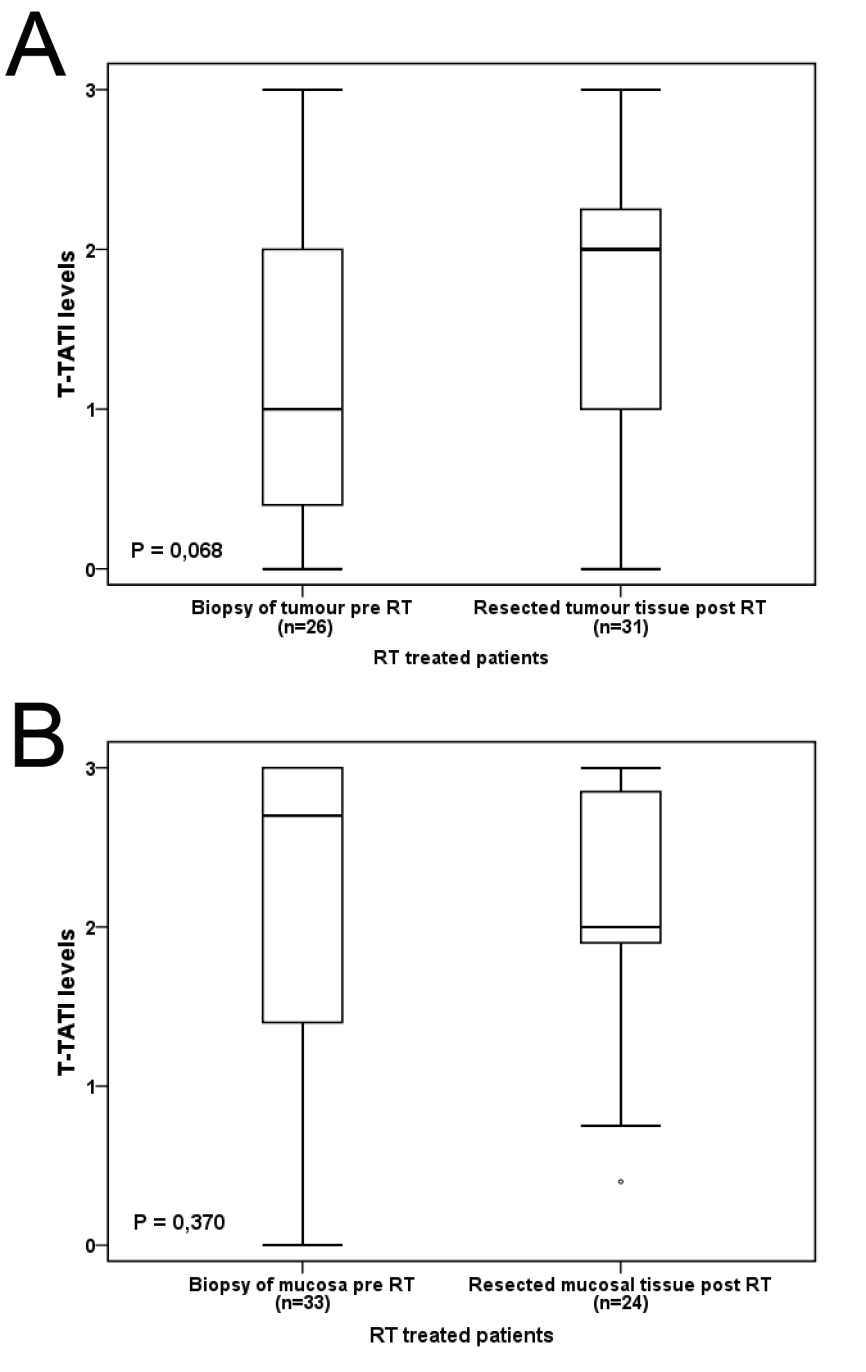
**TATI expression in tumour tissue and normal mucosa before and after radiotherapy**. Box plots showing TATI expression levels in tumour tissue before and after RT (A), and in normal mucosal tissue before and after RT (B), in RT treated patients.

**Figure 2:**
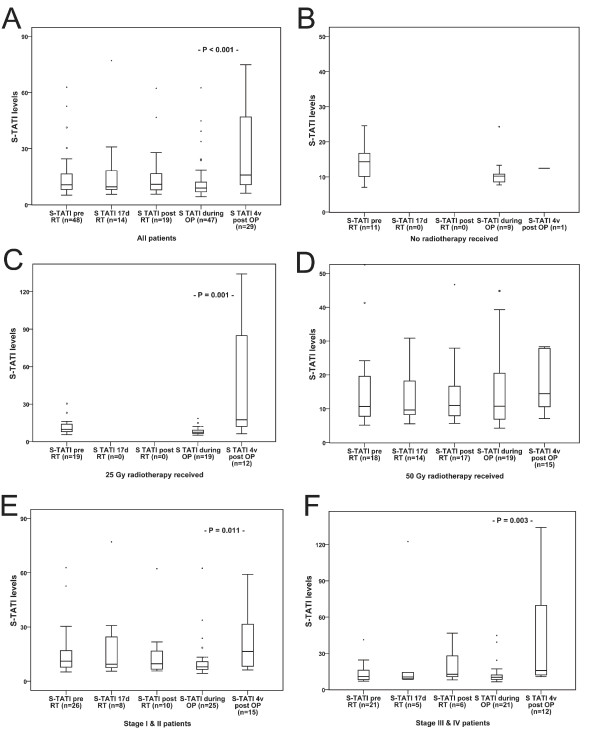
**TATI concentrations in serum at different time points**. Box plots showing s-TATI levels at different time points for; all patients (A), RT subgroups (B-D) and in dichotomized stage subgroups (E-F).

In line with previous findings [1], TATI expression in tumour cells was variable and less abundant than in normal mucosal cells. There was no obvious heterogeneity in t-TATI expression across duplicate cores, which is in line with previous findings [1]. Using a multiplier of fraction and intensity, there was no significant difference between TATI expression in tumour tissue collected prior to RT and after RT (Figure 1A) nor in biopsies taken during RT (n = 8, data not shown), with similar findings in normal mucosa (Figure 1B). In surgically resected tumour specimens, there was no significant difference in t-TATI expression between the different treatment groups (data not shown). There was no significant difference in s-TATI concentrations between samples drawn before, during and after RT in any treatment group (Figure 2A-D). The median s-TATI concentration prior to surgery was 9.06 μg/L (range 4.28-62.49 μg/L). In patients treated with the short-term RT regimen, significantly higher s-TATI concentrations were found in serum drawn post-operatively compared to serum drawn prior to RT or prior to surgery (Z = -3.366, P < 0.001; Figure 2C). In patients treated with long-term RT regimen, there was no significant difference in post-operative s-TATI concentrations compared to earlier time points (P = 0.150). The post-operative increase in s-TATI was higher in patients with Stage III-IV (Z = -2.994, P = 0.003) disease than in with patients with Stage I-II disease (Z = -2.556, P 0.011) (Figure 2E-F). There was no significant difference in s-TATI concentrations in serum drawn at follow-up in patients receiving adjuvant chemotherapy compared to patients not receiving adjuvant chemotherapy (data not shown).

### Association between TATI in tissue and serum and clinicopathological characteristics

T-TATI expression in biopsies extracted before RT did not show any correlation to clinicopathological characteristics; age, gender, disease stage, differentiation or vascular invasion (Table 2). In tumour tissue obtained from surgery, TATI expression correlated significantly with male gender (R = 0.406, P = 0.008) and also with disease stage (R = 0.331, P = 0.033).

**Table 2:** Correlations between t-TATI/s-TATI and clinicopathological characteristics

			t-TATI(f*i)			s-TATI(μg/L)	
		Pre RT		After RT(OP)	Before RT		After RT
**Age at OP**							
R		-0,007		0,114	0,586		0,453
p		0,970		0,474	< 0,001*		0,001*
n		33		42	56		51
							
**Gender**							
R		-0,214		-0,406	0,011		-0,167
p		0,232		0,008*	0,94		0,241
n		33		42	52		51
							
**Disease stage**							
R		0,066		0,330	0,057		0,338
p		0,720		0,033*	0,703		0,021*
n		31		42	47		46
							
**Vascular invasion**							
R		0,204		-0,062	-0,165		-0,053
p		0,262		0,695	0,268		0,725
n		32		42	47		36
							
**s-kreatinin drafted preOP**							
R		0,170		0,038	0,369		0,302
p		0,346		0,812	0,011*		0,041*
n		33		41	47		46

* R: Spearman's correlations coefficient. P < 0.005 n: number of correlated samples.

There was a significant association between age and s-TATI concentrations prior to RT (R = 0.586, P < 0.001) and after RT (R = 0.453, P = 0.001) and a significant association between s-TATI concentrations prior to surgery and a more advanced disease stage (R = 0.338, P = 0.021)(Table 2). In line with previous findings, there was no significant correlation between t-TATI and s-TATI [2]. There was no significant association between s-TATI or t-TATI and s-CEA (data not shown). In general, while t-TATI concentrations in biopsies and in tissue from surgery did not correlate with each other, there was a significant correlation between s-TATI concentrations in samples drawn at the different time points (data not shown). In order to explore whether the association between elevated s-TATI concentrations and increased age can be attributed to an impaired renal function, we analysed the association between s-TATI, and s-creatinine drawn prior to surgery. S-TATI concentrations in serum before RT and prior to surgery showed a modest correlation with s-creatinine (R = 0.369, P = 0.011, R = 0.302, P = 0.041 respectively)(Table 2). There was no significant association between s-TATI at follow-up and s-creatinine (data not shown) and t-TATI expression was not associated to s-creatinine concentrations (data not shown). There was no significant association between age and s-creatinine (data not shown) and no significant difference in t-TATI or s-TATI concentrations in patients treated with adjuvant chemotherapy (n = 13) compared to untreated patients (data not shown).

### Prognostic value of TATI in tissue and serum

**Figure 3:**
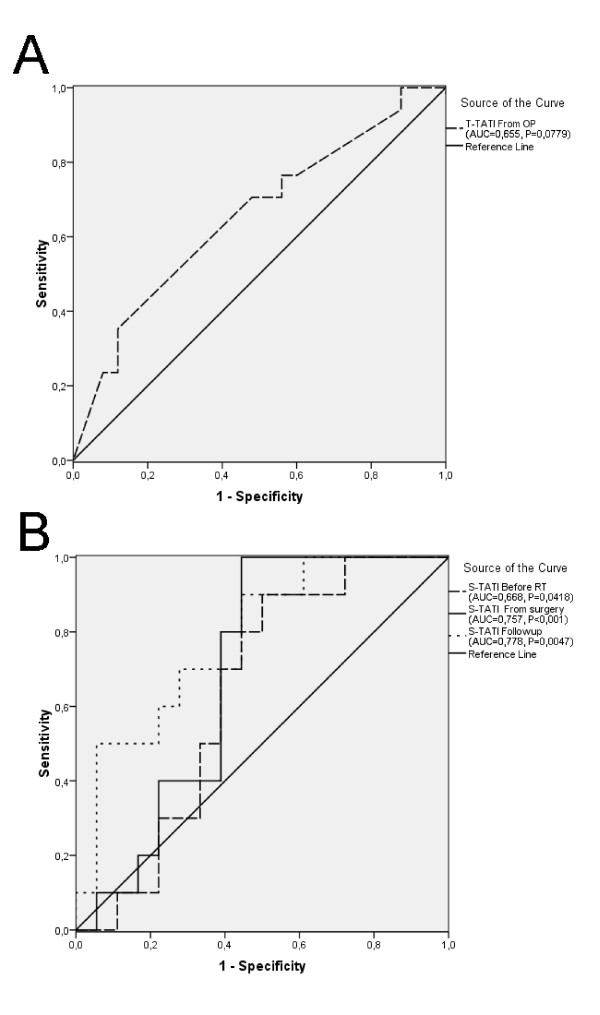
**Prognostic value of TATI concentrations in tumour tissue and serum**. ROC curves showing estimations of the prognostic value of t-TATI in surgically obtained tissue (A) and s-TATI drawn at different time points (B).

**Figure 4:**
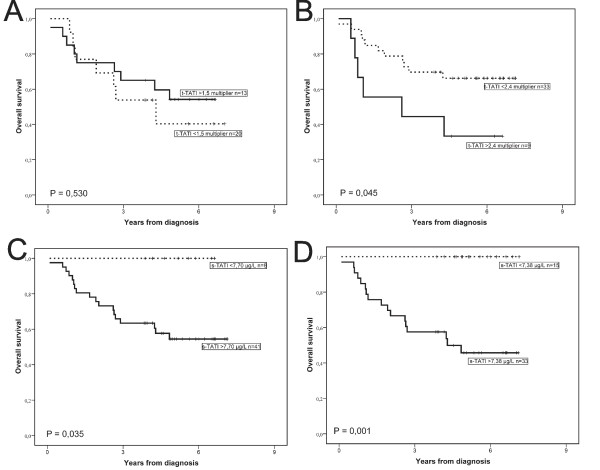
**Kaplan-Meier estimates of overall survival according to TATI concentrations in tumour tissue and serum**. Kaplan-Meier plots showing overall survival according to high and low levels of t-TATI; before RT (A), after RT (B) and for s-TATI; before RT (C), after RT (D).

ROC curve analysis, showed a trend, however non-significant, between higher t-TATI expression (multiplier) and an adverse OS (AUC = 0.655, P = 0.0779; Figure 3A). ROC curve analysis further revealed that the prognostic value of s-TATI was stronger at all time points (before RT; AUC = 0.668, P = 0.0418, prior to surgery; AUC = 0.757, P < 0.001, at follow-up; AUC = 0.777, P = 0.0047; Figure 3B). According to the result of the CRT analysis, a t-TATI fraction-intensity multiplier cut-off at 2.4 was adopted, where levels > 2.4 were considered to denote strong expression (CRT; Additional file 2A). S-TATI cut-offs based on the CRT analysis of preoperatively drawn samples was set to 7.70 μg/L (Additional file 2B), and in serum drawn prior to surgery; 7.38 μg/L (Additional file 2C). The cut-off for s-TATI collected at follow-up was set to 10.74 μg/L, and to 2.6 μg/L for s-CEA. Kaplan-Meier analysis and the log rank test revealed that t-TATI expression in biopsies sampled before RT had no prognostic value (Figure 4A), while a high t-TATI expression in surgical specimens (P = 0.045; Figure 4B) and high s-TATI concentrations before RT (P = 0,035; Figure 4C) and prior to surgery (P = 0.001; Figure 4D), were significantly associated with a shorter OS.

## Discussion

The results from this study show that neoadjuvant RT does not affect TATI concentrations in tissue or serum in rectal cancer patients, and that both, in particular s-TATI, are factors of poor prognosis, further validating previous findings [1,2]. There were no significant differences in t-TATI or s-TATI concentrations in any treatment group, neither when the groups were analysed separately nor when TATI concentrations were compared at different time points; before, during and after treatment. Hence, the previously observed lower s-TATI concentrations in patients with rectal compared to colon cancer patients [2] are unlikely due to effects of neoadjuvant RT. In our previous study, s-TATI concentrations were also found to be significantly higher in right-sided than left-sided colonic cancers [2]. This may indicate that differences in s-TATI concentrations are related to biological characteristics associated with different tumour locations. In contrast, t-TATI expression did not differ according to tumour location [2]. Along this line, as no significant association could be found between t-TATI and s-TATI concentrations neither in this nor in the previous study [2], it could be speculated that TATI concentrations in tumour tissue and serum in CRC patients reflect different biological aspects of the disease.

Further results revealed that t-TATI expression in the surgically resected specimens were significantly higher in males than in females. Interestingly, re-analysis of data from our previous study on t-TATI [1] revealed a significant association between male gender and t-TATI concentrations (n = 105, R = 0.196, P = 0.045) in patients with rectal cancer, but not in patients with colon cancer. Notably, in the present study, there was no significant association between TATI expression in tumour biopsies sampled before RT and gender, but this was possibly due to the smaller number of cases available for analysis. These results could however also reflect a more representative sampling of tissue from the surgically resected specimens. Discrepant immunohistochemical staining results between biopsies and full tissue sections have been reported [16].

As male gender is associated with an increased mortality from colorectal cancer [17], and TATI has been associated with a more aggressive tumour phenotype [13], it could be speculated that males to a larger extent have more aggressive tumour forms, with higher TATI expression. This hypothesis does however not explain why s-TATI concentrations were not associated with gender, neither in the present nor in our previous study [2], and in both studies, s-TATI was a stronger prognostic factor than t-TATI.

There were significant associations between s-TATI, but not t-TATI, and age at diagnosis, in serum drawn at all time points in this study, which is in line with our previous findings [2]. As we found a significant association between higher s-TATI concentrations and s-creatinine, this could in part be explained by an impaired renal function in elderly patients, which is in line with previous findings demonstrating an association between increased s-TATI concentrations and an impaired renal function [18]. Notably, s-creatinine concentration levels were not available for the patients included in the previous study on s-TATI [2], and although the sample size in this study is too small to draw any firm conclusions, s-creatinine should be taken into consideration in future studies on the role of s-TATI as a prognostic biomarker in CRC. For s-TATI, the optimal cut-offs derived from CRT analyses were identical to optimal cut-offs according to ROC curve analyses and s-TATI remained prognostic also when higher cut-offs were used in the survival analyses. The median concentration level of s-TATI was lower in the present study (9.06 μg/L) than in the previous study on colorectal cancer patients (median; 13.42 μg/L), where colon cancer patients had significantly higher s-TATI levels (14.62 μg/L) than rectal cancer patients (median; 12.48 μg/L)[2]. Hence, the cut-offs derived from CRT analysis were slightly lower in this study.

Higher TATI concentrations in serum drawn prior to surgery correlated with disease stage, which is in line with our previous study [2]. There was also a significant association between higher t-TATI expression in the surgical specimens, but not in biopsies, and a more advanced disease stage. In our previous study there was no significant association between t-TATI and clinical stage, neither in the full cohort nor in rectal cancer patients [1]. The lack of an association between t-TATI and s-TATI with s-CEA concentrations is also in line with previous findings [2]. We are not aware of any other studies on the effect of RT on TATI concentrations in tissue or serum, but studies on another protease inhibitor; tissue inhibitor of metalloproteinase 1 (TIMP-1), have shown that the expression in tumour tissue is unaffected by RT [5,19]. However, plasma concentrations of TIMP-1 have been found to increase after combined RT-chemotherapy treatment [20]. Interestingly, we found a significant post-operative increase in s-TATI concentrations in patients treated by short-term RT but not in long-term RT treated patients. In line with previous findings, s-TATI concentrations increased considerably after surgery for many of the short-term regimen treated patients, which supports the theory that TATI can behave as an acute phase reactant, as demonstrated by Solakidi et al. [21].

Similarly, in our study s-TATI concentrations remained largely unaffected throughout RT until surgery in both short-term and long-term RT treated patients, and the elevated concentrations in short term RT treated patients were only seen 4 weeks post-surgery.

We did not find any significant differences in s-TATI concentrations according to adjuvant chemotherapy and there was no difference in the distribution of clinicopathological characteristics in patients receiving RT and untreated patients, decreasing the probability of a patient selection bias.

## Conclusions

In this study, we demonstrate that concentrations of TATI in tumour tissue or serum are not affected by neoadjuvant radiotherapy in rectal cancer patients. The finding of an association between both t-TATI and s-TATI, in particular the latter, and an impaired survival is in line with previous results, and further supports the potential utility of TATI as a prognostic biomarker in patients with cancer of the colon and rectum, irrespective of neoadjuvant RT.

## Competing interests

The authors declare that they have no competing interests.

## Authors' contributions

AG participated in the collection of data, performed statistical analyses and drafted the manuscript. CS participated in the collection of tissue samples, data and revised the manuscript. KH performed the serum analyses and revised the manuscript. IP assisted in the collection of tissue samples and data. AB participated in the conception of the study, BN assisted with the TMA construction and revision of the manuscript, UHS participated in revision of the manuscript, BJ participated in the conception and design of the study, LBJ participated in the collection of tissue samples, data and revised the manuscript, KJ participated in the conception and design of the study, statistical analysis, drafted and revised the manuscript. All authors read and approved the final manuscript.

## Supplementary Material

Additional file 1 **Study design**. Flowchart illustrating treatment course and sample collection in subgroups according to neoadjuvant radio therapy. Click here for file

Additional file 2 **Classification regression tree charts**. Classification regression trees of t-TATI expression in resected specimen (A), s-TATI drawn before RT (B) and at surgery (C). Click here for file
