# Supplementary material for: Effects of radiation therapy on tissue and serum concentrations of tumour associated trypsin inhibitor and their prognostic significance in rectal cancer patients
Source: Radiat Oncol. 2011 Aug 24;6:100. doi: 10.1186/1748-717X-6-100 (PMC3173337; doi:10.1186/1748-717X-6-100)
Supplement: Additional file 1 — Study design. Flowchart illustrating treatment course and sample collection in subgroups according to neoadjuvant radio therapy. [file 1748-717X-6-100-S1.PDF]

Patients receiving no RT

Inclusion<br>Serum<br>Biopsy	4-6 weeks respite	Surgery<br>Serum<br>Resected tumour	4 weeks respite	Follow-up<br>Serum

Patients receiving short-term RT

Pre RT<br>Serum<br>Biopsy	Radiotherapy course<br>25 Gy / 5 days	3 days respite	Surgery<br>Serum<br>Resected tumour	4 weeks respite	Follow-up<br>Serum

Patients receiving long-term RT

Pre RT<br>Serum<br>Biopsy	Radiotherapy course 50 Gy / 5 weeks<br>Serum<br>Biopsy	Post RT<br>Serum<br>Biopsy	4-6 weeks respite	Surgery<br>Serum<br>Resected tumour	4 weeks respite	Follow-up<br>Serum
